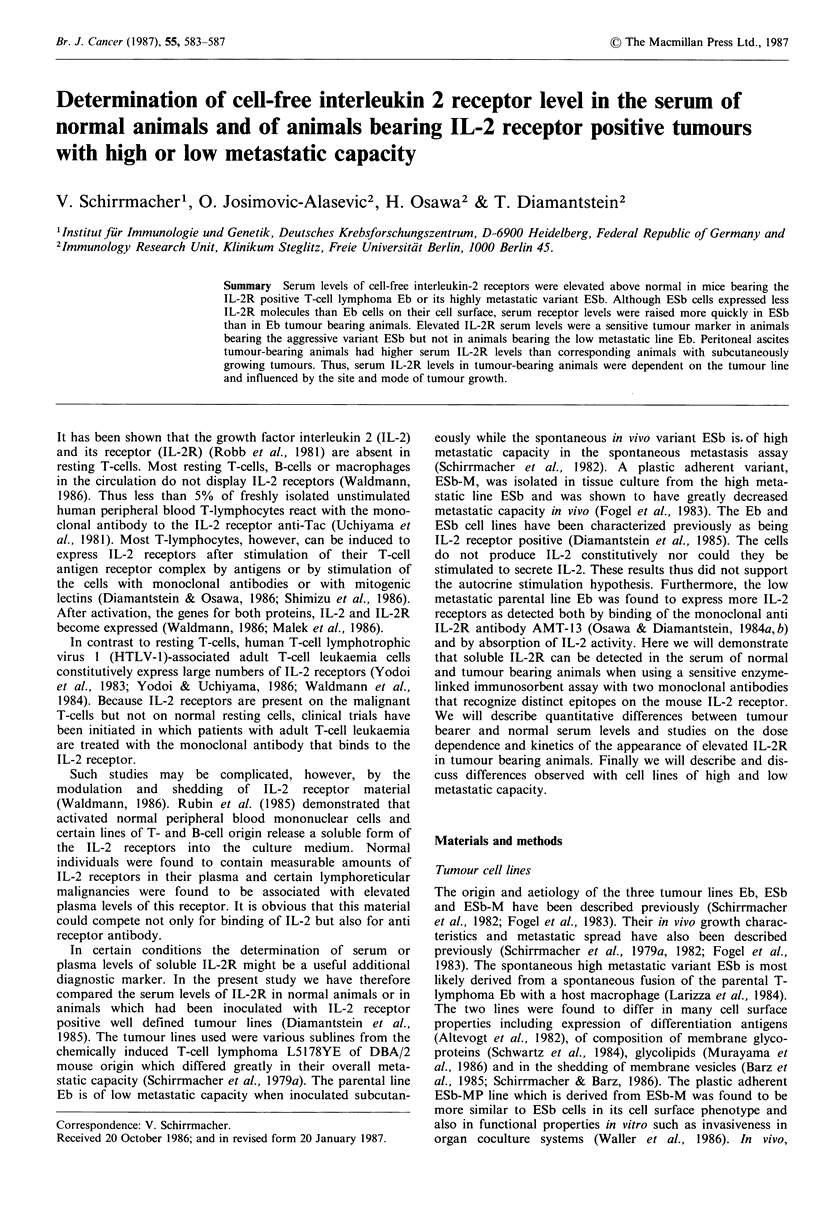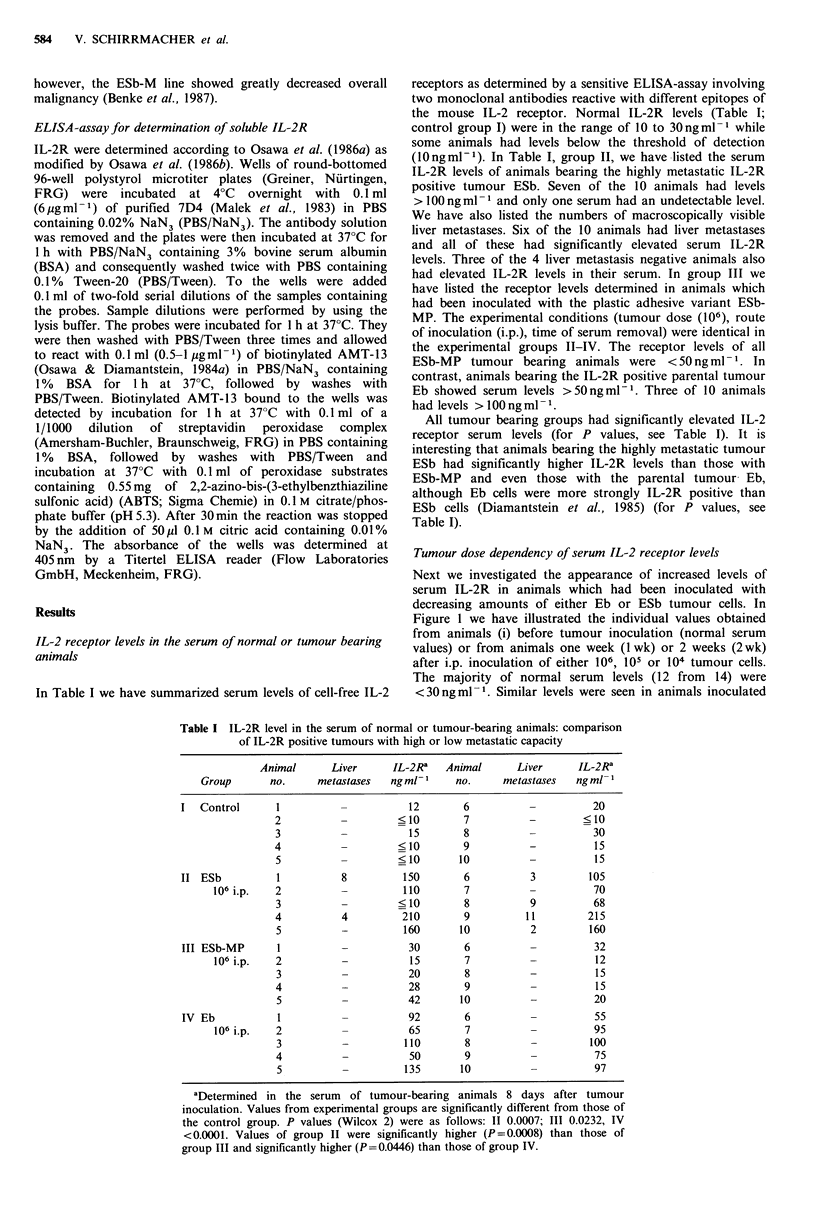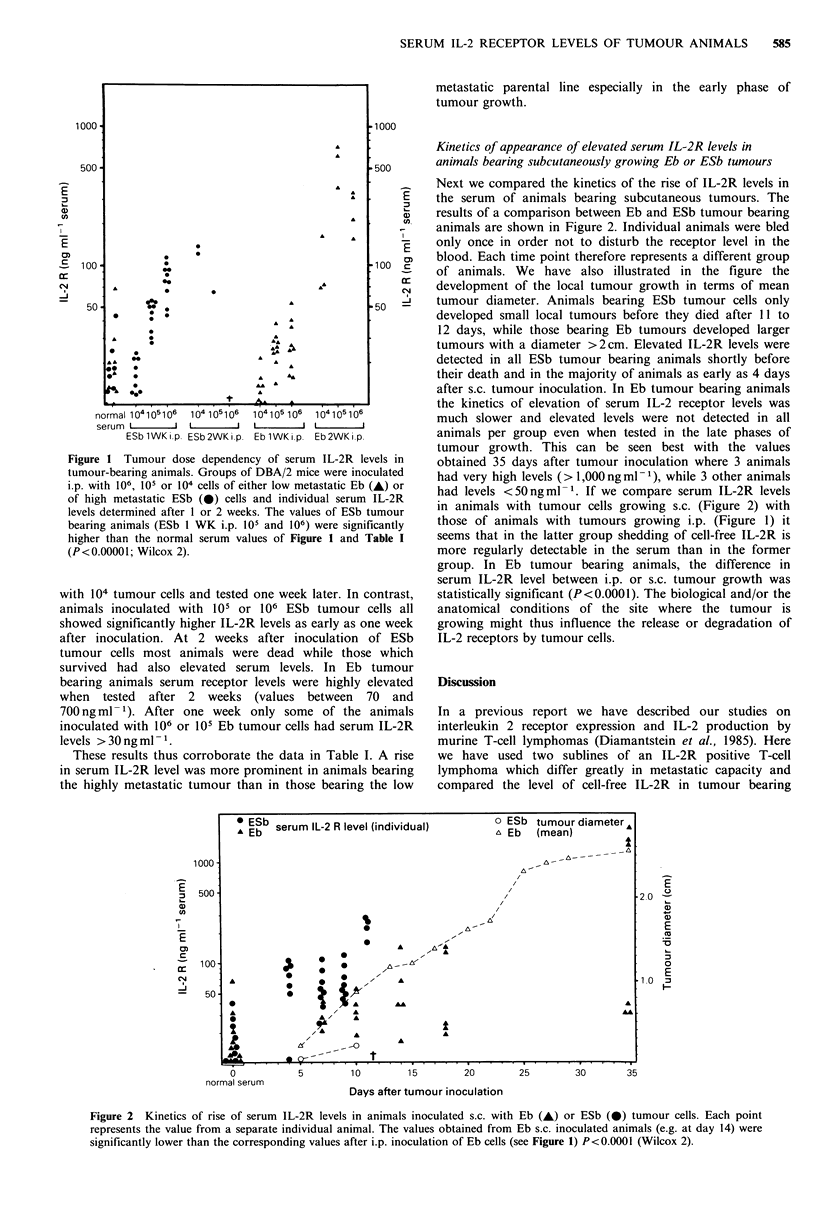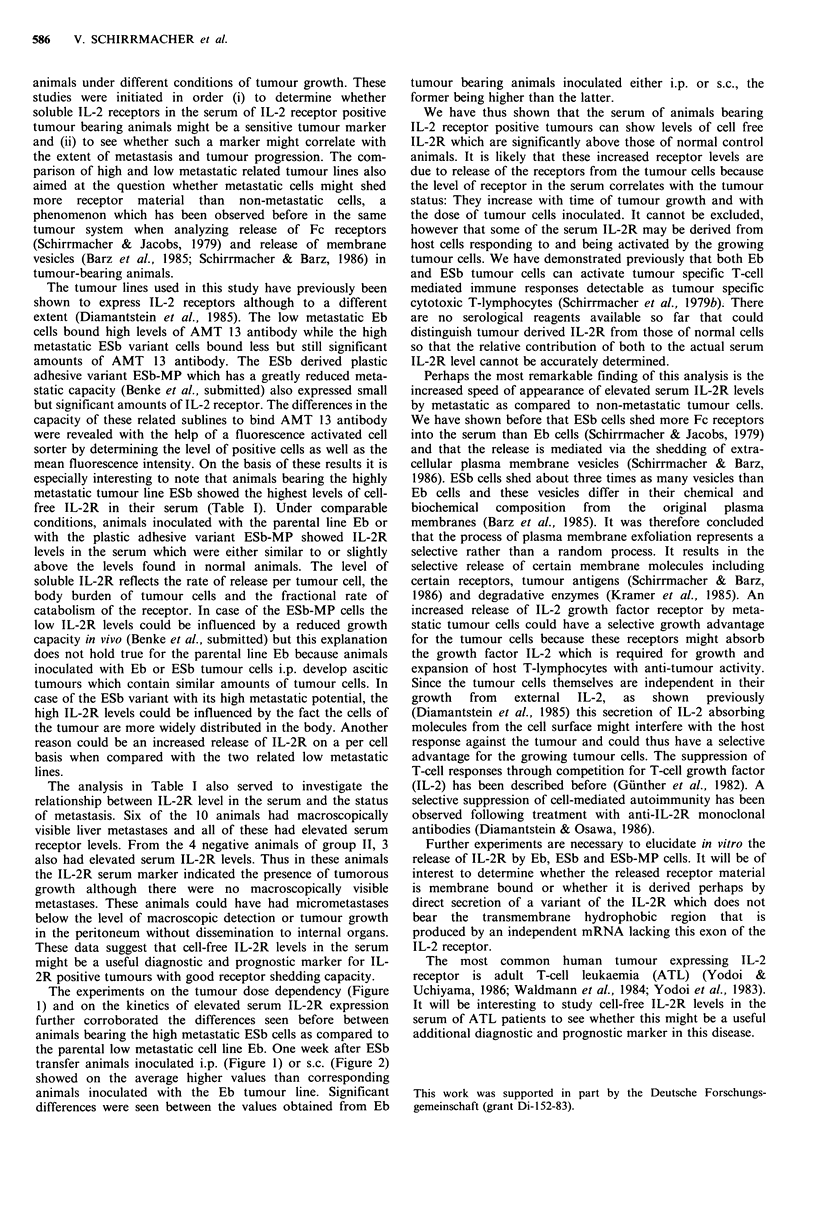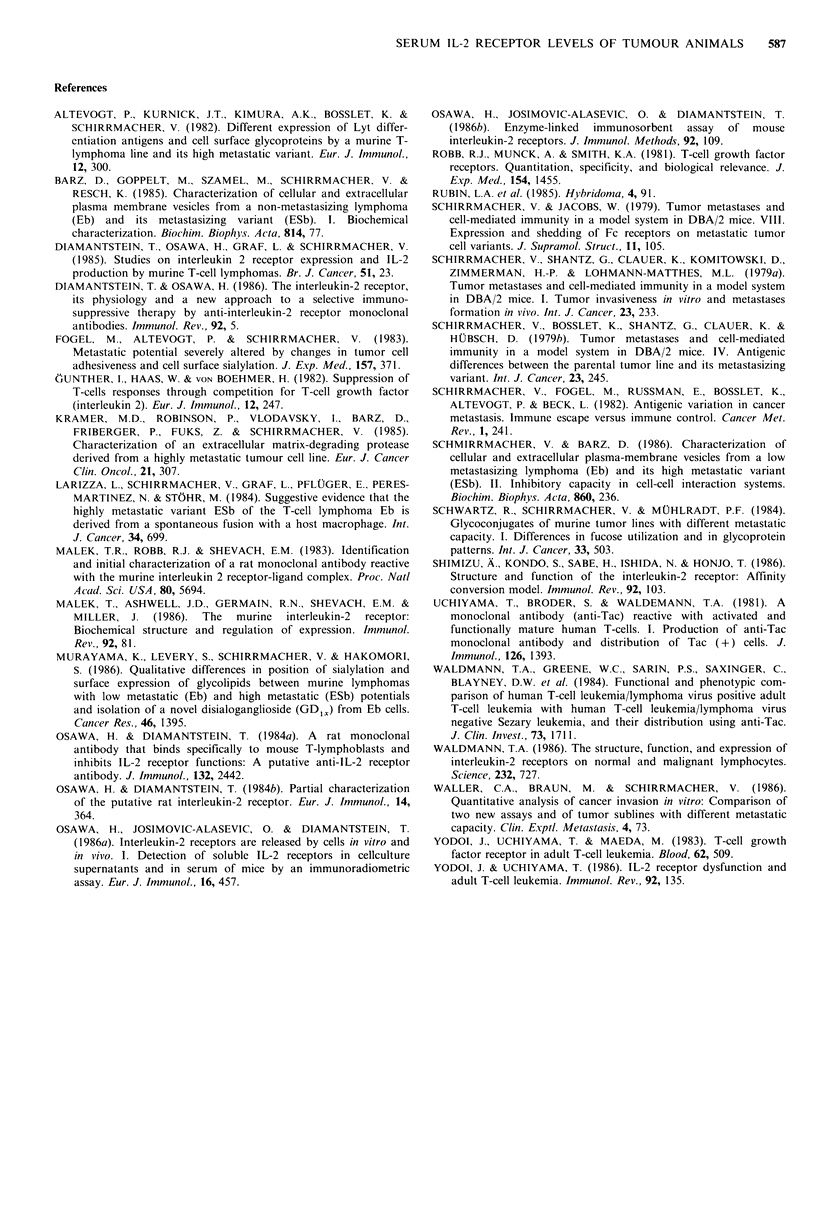# Determination of cell-free interleukin 2 receptor level in the serum of normal animals and of animals bearing IL-2 receptor positive tumours with high or low metastatic capacity.

**DOI:** 10.1038/bjc.1987.119

**Published:** 1987-06

**Authors:** V. Schirrmacher, O. Josimovic-Alasevic, H. Osawa, T. Diamantstein

## Abstract

Serum levels of cell-free interleukin-2 receptors were elevated above normal in mice bearing the IL-2R positive T-cell lymphoma Eb or its highly metastatic variant ESb. Although ESb cells expressed less IL-2R molecules than Eb cells on their cell surface, serum receptor levels were raised more quickly in ESb than in Eb tumour bearing animals. Elevated IL-2R serum levels were a sensitive tumour marker in animals bearing the aggressive variant ESb but not in animals bearing the low metastatic line Eb. Peritoneal ascites tumour-bearing animals had higher serum IL-2R levels than corresponding animals with subcutaneously growing tumours. Thus, serum IL-2R levels in tumour-bearing animals were dependent on the tumour line and influenced by the site and mode of tumour growth.


*Br. J. Cancer* (1987), **55**, 583–587 © The Macmillan Press Ltd., 1987

### Determination of cell-free interleukin 2 receptor level in the serum of normal animals and of animals bearing IL-2 receptor positive tumours with high or low metastatic capacity

V. Schirrmacher[1], O. Josimovic-Alasevic[2], H. Osawa[2] & T. Diamantstein[2]

[1]*Institut für Immunologie und Genetik, Deutsches Krebsforschungszentrum, D-6900 Heidelberg, Federal Republic of Germany and* [2]*Immunology Research Unit, Klinikum Steglitz, Freie Universität Berlin, 1000 Berlin 45.*

**Summary** Serum levels of cell-free interleukin-2 receptors were elevated above normal in mice bearing the IL-2R positive T-cell lymphoma Eb or its highly metastatic variant ESb. Although ESb cells expressed less IL-2R molecules than Eb cells on their cell surface, serum receptor levels were raised more quickly in ESb than in Eb tumour bearing animals. Elevated IL-2R serum levels were a sensitive tumour marker in animals bearing the aggressive variant ESb but not in animals bearing the low metastatic line Eb. Peritoneal ascites tumour-bearing animals had higher serum IL-2R levels than corresponding animals with subcutaneously growing tumours. Thus, serum IL-2R levels in tumour-bearing animals were dependent on the tumour line and influenced by the site and mode of tumour growth.

It has been shown that the growth factor interleukin 2 (IL-2) and its receptor (IL-2R) (Robb *et al.*, 1981) are absent in resting T-cells. Most resting T-cells, B-cells or macrophages in the circulation do not display IL-2 receptors (Waldmann, 1986). Thus less than 5% of freshly isolated unstimulated human peripheral blood T-lymphocytes react with the monoclonal antibody to the IL-2 receptor anti-Tac (Uchiyama *et al.*, 1981). Most T-lymphocytes, however, can be induced to express IL-2 receptors after stimulation of their T-cell antigen receptor complex by antigens or by stimulation of the cells with monoclonal antibodies or with mitogenic lectins (Diamantstein & Osawa, 1986; Shimizu *et al.*, 1986). After activation, the genes for both proteins, IL-2 and IL-2R become expressed (Waldmann, 1986; Malek *et al.*, 1986).

In contrast to resting T-cells, human T-cell lymphotrophic virus 1 (HTLV-1)-associated adult T-cell leukaemia cells constitutively express large numbers of IL-2 receptors (Yodoi *et al.*, 1983; Yodoi & Uchiyama, 1986; Waldmann *et al.*, 1984). Because IL-2 receptors are present on the malignant T-cells but not on normal resting cells, clinical trials have been initiated in which patients with adult T-cell leukaemia are treated with the monoclonal antibody that binds to the IL-2 receptor.

Such studies may be complicated, however, by the modulation and shedding of IL-2 receptor material (Waldmann, 1986). Rubin *et al.* (1985) demonstrated that activated normal peripheral blood mononuclear cells and certain lines of T- and B-cell origin release a soluble form of the IL-2 receptors into the culture medium. Normal individuals were found to contain measurable amounts of IL-2 receptors in their plasma and certain lymphoreticular malignancies were found to be associated with elevated plasma levels of this receptor. It is obvious that this material could compete not only for binding of IL-2 but also for anti receptor antibody.

In certain conditions the determination of serum or plasma levels of soluble IL-2R might be a useful additional diagnostic marker. In the present study we have therefore compared the serum levels of IL-2R in normal animals or in animals which had been inoculated with IL-2 receptor positive well defined tumour lines (Diamantstein *et al.*, 1985). The tumour lines used were various sublines from the chemically induced T-cell lymphoma L5178YE of DBA/2 mouse origin which differed greatly in their overall metastatic capacity (Schirrmacher *et al.*, 1979*a*). The parental line Eb is of low metastatic capacity when inoculated subcutaneously while the spontaneous *in vivo* variant ESb is of high metastatic capacity in the spontaneous metastasis assay (Schirrmacher *et al.*, 1982). A plastic adherent variant, ESb-M, was isolated in tissue culture from the high metastatic line ESb and was shown to have greatly decreased metastatic capacity *in vivo* (Fogel *et al.*, 1983). The Eb and ESb cell lines have been characterized previously as being IL-2 receptor positive (Diamantstein *et al.*, 1985). The cells do not produce IL-2 constitutively nor could they be stimulated to secrete IL-2. These results thus did not support the autocrine stimulation hypothesis. Furthermore, the low metastatic parental line Eb was found to express more IL-2 receptors as detected both by binding of the monoclonal anti IL-2R antibody AMT-13 (Osawa & Diamantstein, 1984*a*,*b*) and by absorption of IL-2 activity. Here we will demonstrate that soluble IL-2R can be detected in the serum of normal and tumour bearing animals when using a sensitive enzyme-linked immunosorbent assay with two monoclonal antibodies that recognize distinct epitopes on the mouse IL-2 receptor. We will describe quantitative differences between tumour bearer and normal serum levels and studies on the dose dependence and kinetics of the appearance of elevated IL-2R in tumour bearing animals. Finally we will describe and discuss differences observed with cell lines of high and low metastatic capacity.

### Materials and methods

### *Tumour cell lines*

The origin and aetiology of the three tumour lines Eb, ESb and ESb-M have been described previously (Schirrmacher *et al.*, 1982; Fogel *et al.*, 1983). Their *in vivo* growth characteristics and metastatic spread have also been described previously (Schirrmacher *et al.*, 1979*a*, 1982; Fogel *et al.*, 1983). The spontaneous high metastatic variant ESb is most likely derived from a spontaneous fusion of the parental T-lymphoma Eb with a host macrophage (Larizza *et al.*, 1984). The two lines were found to differ in many cell surface properties including expression of differentiation antigens (Altevogt *et al.*, 1982), of composition of membrane glycoproteins (Schwartz *et al.*, 1984), glycolipids (Murayama *et al.*, 1986) and in the shedding of membrane vesicles (Barz *et al.*, 1985; Schirrmacher & Barz, 1986). The plastic adherent ESb-MP line which is derived from ESb-M was found to be more similar to ESb cells in its cell surface phenotype and also in functional properties *in vitro* such as invasiveness in organ coculture systems (Waller *et al.*, 1986). *In vivo*,

Correspondence: V. Schirrmacher.
Received 20 October 1986; and in revised form 20 January 1987.

however, the ESb-M line showed greatly decreased overall malignancy (Benke *et al.*, 1987).

*ELISA-assay for determination of soluble IL-2R*

IL-2R were determined according to Osawa *et al.* (1986*a*) as modified by Osawa *et al.* (1986*b*). Wells of round-bottomed 96-well polystyrol microtiter plates (Greiner, Nürtingen, FRG) were incubated at 4°C overnight with 0.1 ml (6 $\mu g ml^{-1}$) of purified 7D4 (Malek *et al.*, 1983) in PBS containing 0.02% $NaN_3$ (PBS/$NaN_3$). The antibody solution was removed and the plates were then incubated at 37°C for 1 h with PBS/$NaN_3$ containing 3% bovine serum albumin (BSA) and consequently washed twice with PBS containing 0.1% Tween-20 (PBS/Tween). To the wells were added 0.1 ml of two-fold serial dilutions of the samples containing the probes. Sample dilutions were performed by using the lysis buffer. The probes were incubated for 1 h at 37°C. They were then washed with PBS/Tween three times and allowed to react with 0.1 ml (0.5–1 $\mu g ml^{-1}$) of biotinylated AMT-13 (Osawa & Diamantstein, 1984*a*) in PBS/$NaN_3$ containing 1% BSA for 1 h at 37°C, followed by washes with PBS/Tween. Biotinylated AMT-13 bound to the wells was detected by incubation for 1 h at 37°C with 0.1 ml of a 1/1000 dilution of streptavidin peroxidase complex (Amersham-Buchler, Braunschweig, FRG) in PBS containing 1% BSA, followed by washes with PBS/Tween and incubation at 37°C with 0.1 ml of peroxidase substrates containing 0.55 mg of 2,2-azino-bis-(3-ethylbenzthiaziline sulfonic acid) (ABTS; Sigma Chemie) in 0.1 M citrate/phosphate buffer (pH 5.3). After 30 min the reaction was stopped by the addition of 50 $\mu l$ 0.1 M citric acid containing 0.01% $NaN_3$. The absorbance of the wells was determined at 405 nm by a Titertel ELISA reader (Flow Laboratories GmbH, Meckenheim, FRG).

### Results

*IL-2 receptor levels in the serum of normal or tumour bearing animals*

In Table I we have summarized serum levels of cell-free IL-2 receptors as determined by a sensitive ELISA-assay involving two monoclonal antibodies reactive with different epitopes of the mouse IL-2 receptor. Normal IL-2R levels (Table I; control group I) were in the range of 10 to 30 $ng ml^{-1}$ while some animals had levels below the threshold of detection (10 $ng ml^{-1}$). In Table I, group II, we have listed the serum IL-2R levels of animals bearing the highly metastatic IL-2R positive tumour ESb. Seven of the 10 animals had levels $>100 ng ml^{-1}$ and only one serum had an undetectable level. We have also listed the numbers of macroscopically visible liver metastases. Six of the 10 animals had liver metastases and all of these had significantly elevated serum IL-2R levels. Three of the 4 liver metastasis negative animals also had elevated IL-2R levels in their serum. In group III we have listed the receptor levels determined in animals which had been inoculated with the plastic adhesive variant ESb-MP. The experimental conditions (tumour dose ($10^6$), route of inoculation (i.p.), time of serum removal) were identical in the experimental groups II–IV. The receptor levels of all ESb-MP tumour bearing animals were $<50 ng ml^{-1}$. In contrast, animals bearing the IL-2R positive parental tumour Eb showed serum levels $>50 ng ml^{-1}$. Three of 10 animals had levels $>100 ng ml^{-1}$.

All tumour bearing groups had significantly elevated IL-2 receptor serum levels (for *P* values, see Table I). It is interesting that animals bearing the highly metastatic tumour ESb had significantly higher IL-2R levels than those with ESb-MP and even those with the parental tumour Eb, although Eb cells were more strongly IL-2R positive than ESb cells (Diamantstein *et al.*, 1985) (for *P* values, see Table I).

*Tumour dose dependency of serum IL-2 receptor levels*

Next we investigated the appearance of increased levels of serum IL-2R in animals which had been inoculated with decreasing amounts of either Eb or ESb tumour cells. In Figure 1 we have illustrated the individual values obtained from animals (i) before tumour inoculation (normal serum values) or from animals one week (1 wk) or 2 weeks (2 wk) after i.p. inoculation of either $10^6$, $10^5$ or $10^4$ tumour cells. The majority of normal serum levels (12 from 14) were $<30 ng ml^{-1}$. Similar levels were seen in animals inoculated

**Table I** IL-2R level in the serum of normal or tumour-bearing animals: comparison of IL-2R positive tumours with high or low metastatic capacity

| Group | *Animal no.* | *Liver metastases* | *IL-2R*[a] *ng ml*$^{-1}$ | *Animal no.* | *Liver metastases* | *IL-2R*[a] *ng ml*$^{-1}$ |
|---|---|---|---|---|---|---|
| I Control | 1 | – | 12 | 6 | – | 20 |
| | 2 | – | ≦10 | 7 | – | ≦10 |
| | 3 | – | 15 | 8 | – | 30 |
| | 4 | – | ≦10 | 9 | – | 15 |
| | 5 | – | ≦10 | 10 | – | 15 |
| II ESb | 1 | 8 | 150 | 6 | 3 | 105 |
| $10^6$ i.p. | 2 | – | 110 | 7 | – | 70 |
| | 3 | – | ≦10 | 8 | 9 | 68 |
| | 4 | 4 | 210 | 9 | 11 | 215 |
| | 5 | – | 160 | 10 | 2 | 160 |
| III ESb-MP | 1 | – | 30 | 6 | – | 32 |
| $10^6$ i.p. | 2 | – | 15 | 7 | – | 12 |
| | 3 | – | 20 | 8 | – | 15 |
| | 4 | – | 28 | 9 | – | 15 |
| | 5 | – | 42 | 10 | – | 20 |
| IV Eb | 1 | – | 92 | 6 | – | 55 |
| $10^6$ i.p. | 2 | – | 65 | 7 | – | 95 |
| | 3 | – | 110 | 8 | – | 100 |
| | 4 | – | 50 | 9 | – | 75 |
| | 5 | – | 135 | 10 | – | 97 |

[a]Determined in the serum of tumour-bearing animals 8 days after tumour inoculation. Values from experimental groups are significantly different from those of the control group. *P* values (Wilcox 2) were as follows: II 0.0007; III 0.0232, IV <0.0001. Values of group II were significantly higher ($P=0.0008$) than those of group III and significantly higher ($P=0.0446$) than those of group IV.

**Figure 1** Tumour dose dependency of serum IL-2R levels in tumour-bearing animals. Groups of DBA/2 mice were inoculated i.p. with $10^6$, $10^5$ or $10^4$ cells of either low metastatic Eb (▲) or of high metastatic ESb (●) cells and individual serum IL-2R levels determined after 1 or 2 weeks. The values of ESb tumour bearing animals (ESb 1 WK i.p. $10^5$ and $10^6$) were significantly higher than the normal serum values of **Figure 1** and **Table I** ($P<0.00001$; Wilcox 2).

with $10^4$ tumour cells and tested one week later. In contrast, animals inoculated with $10^5$ or $10^6$ ESb tumour cells all showed significantly higher IL-2R levels as early as one week after inoculation. At 2 weeks after inoculation of ESb tumour cells most animals were dead while those which survived had also elevated serum levels. In Eb tumour bearing animals serum receptor levels were highly elevated when tested after 2 weeks (values between 70 and 700 ng ml$^{-1}$). After one week only some of the animals inoculated with $10^6$ or $10^5$ Eb tumour cells had serum IL-2R levels $>30$ ng ml$^{-1}$.

These results thus corroborate the data in Table I. A rise in serum IL-2R level was more prominent in animals bearing the highly metastatic tumour than in those bearing the low metastatic parental line especially in the early phase of tumour growth.

*Kinetics of appearance of elevated serum IL-2R levels in animals bearing subcutaneously growing Eb or ESb tumours*

Next we compared the kinetics of the rise of IL-2R levels in the serum of animals bearing subcutaneous tumours. The results of a comparison between Eb and ESb tumour bearing animals are shown in Figure 2. Individual animals were bled only once in order not to disturb the receptor level in the blood. Each time point therefore represents a different group of animals. We have also illustrated in the figure the development of the local tumour growth in terms of mean tumour diameter. Animals bearing ESb tumour cells only developed small local tumours before they died after 11 to 12 days, while those bearing Eb tumours developed larger tumours with a diameter $>2$ cm. Elevated IL-2R levels were detected in all ESb tumour bearing animals shortly before their death and in the majority of animals as early as 4 days after s.c. tumour inoculation. In Eb tumour bearing animals the kinetics of elevation of serum IL-2 receptor levels was much slower and elevated levels were not detected in all animals per group even when tested in the late phases of tumour growth. This can be seen best with the values obtained 35 days after tumour inoculation where 3 animals had very high levels ($>1,000$ ng ml$^{-1}$), while 3 other animals had levels $<50$ ng ml$^{-1}$. If we compare serum IL-2R levels in animals with tumour cells growing s.c. (Figure 2) with those of animals with tumours growing i.p. (Figure 1) it seems that in the latter group shedding of cell-free IL-2R is more regularly detectable in the serum than in the former group. In Eb tumour bearing animals, the difference in serum IL-2R level between i.p. or s.c. tumour growth was statistically significant ($P<0.0001$). The biological and/or the anatomical conditions of the site where the tumour is growing might thus influence the release or degradation of IL-2 receptors by tumour cells.

### Discussion

In a previous report we have described our studies on interleukin 2 receptor expression and IL-2 production by murine T-cell lymphomas (Diamantstein *et al.*, 1985). Here we have used two sublines of an IL-2R positive T-cell lymphoma which differ greatly in metastatic capacity and compared the level of cell-free IL-2R in tumour bearing

**Figure 2** Kinetics of rise of serum IL-2R levels in animals inoculated s.c. with Eb (▲) or ESb (●) tumour cells. Each point represents the value from a separate individual animal. The values obtained from Eb s.c. inoculated animals (e.g. at day 14) were significantly lower than the corresponding values after i.p. inoculation of Eb cells (see **Figure 1**) $P<0.0001$ (Wilcox 2).

animals under different conditions of tumour growth. These studies were initiated in order (i) to determine whether soluble IL-2 receptors in the serum of IL-2 receptor positive tumour bearing animals might be a sensitive tumour marker and (ii) to see whether such a marker might correlate with the extent of metastasis and tumour progression. The comparison of high and low metastatic related tumour lines also aimed at the question whether metastatic cells might shed more receptor material than non-metastatic cells, a phenomenon which has been observed before in the same tumour system when analyzing release of Fc receptors (Schirrmacher & Jacobs, 1979) and release of membrane vesicles (Barz *et al.*, 1985; Schirrmacher & Barz, 1986) in tumour-bearing animals.

The tumour lines used in this study have previously been shown to express IL-2 receptors although to a different extent (Diamantstein *et al.*, 1985). The low metastatic Eb cells bound high levels of AMT 13 antibody while the high metastatic ESb variant cells bound less but still significant amounts of AMT 13 antibody. The ESb derived plastic adhesive variant ESb-MP which has a greatly reduced metastatic capacity (Benke *et al.*, submitted) also expressed small but significant amounts of IL-2 receptor. The differences in the capacity of these related sublines to bind AMT 13 antibody were revealed with the help of a fluorescence activated cell sorter by determining the level of positive cells as well as the mean fluorescence intensity. On the basis of these results it is especially interesting to note that animals bearing the highly metastatic tumour line ESb showed the highest levels of cell-free IL-2R in their serum (Table I). Under comparable conditions, animals inoculated with the parental line Eb or with the plastic adhesive variant ESb-MP showed IL-2R levels in the serum which were either similar to or slightly above the levels found in normal animals. The level of soluble IL-2R reflects the rate of release per tumour cell, the body burden of tumour cells and the fractional rate of catabolism of the receptor. In case of the ESb-MP cells the low IL-2R levels could be influenced by a reduced growth capacity *in vivo* (Benke *et al.*, submitted) but this explanation does not hold true for the parental line Eb because animals inoculated with Eb or ESb tumour cells i.p. develop ascitic tumours which contain similar amounts of tumour cells. In case of the ESb variant with its high metastatic potential, the high IL-2R levels could be influenced by the fact the cells of the tumour are more widely distributed in the body. Another reason could be an increased release of IL-2R on a per cell basis when compared with the two related low metastatic lines.

The analysis in Table I also served to investigate the relationship between IL-2R level in the serum and the status of metastasis. Six of the 10 animals had macroscopically visible liver metastases and all of these had elevated serum receptor levels. From the 4 negative animals of group II, 3 also had elevated serum IL-2R levels. Thus in these animals the IL-2R serum marker indicated the presence of tumorous growth although there were no macroscopically visible metastases. These animals could have had micrometastases below the level of macroscopic detection or tumour growth in the peritoneum without dissemination to internal organs. These data suggest that cell-free IL-2R levels in the serum might be a useful diagnostic and prognostic marker for IL-2R positive tumours with good receptor shedding capacity.

The experiments on the tumour dose dependency (Figure 1) and on the kinetics of elevated serum IL-2R expression further corroborated the differences seen before between animals bearing the high metastatic ESb cells as compared to the parental low metastatic cell line Eb. One week after ESb transfer animals inoculated i.p. (Figure 1) or s.c. (Figure 2) showed on the average higher values than corresponding animals inoculated with the Eb tumour line. Significant differences were seen between the values obtained from Eb tumour bearing animals inoculated either i.p. or s.c., the former being higher than the latter.

We have thus shown that the serum of animals bearing IL-2 receptor positive tumours can show levels of cell free IL-2R which are significantly above those of normal control animals. It is likely that these increased receptor levels are due to release of the receptors from the tumour cells because the level of receptor in the serum correlates with the tumour status: They increase with time of tumour growth and with the dose of tumour cells inoculated. It cannot be excluded, however that some of the serum IL-2R may be derived from host cells responding to and being activated by the growing tumour cells. We have demonstrated previously that both Eb and ESb tumour cells can activate tumour specific T-cell mediated immune responses detectable as tumour specific cytotoxic T-lymphocytes (Schirrmacher *et al.*, 1979*b*). There are no serological reagents available so far that could distinguish tumour derived IL-2R from those of normal cells so that the relative contribution of both to the actual serum IL-2R level cannot be accurately determined.

Perhaps the most remarkable finding of this analysis is the increased speed of appearance of elevated serum IL-2R levels by metastatic as compared to non-metastatic tumour cells. We have shown before that ESb cells shed more Fc receptors into the serum than Eb cells (Schirrmacher & Jacobs, 1979) and that the release is mediated via the shedding of extracellular plasma membrane vesicles (Schirrmacher & Barz, 1986). ESb cells shed about three times as many vesicles than Eb cells and these vesicles differ in their chemical and biochemical composition from the original plasma membranes (Barz *et al.*, 1985). It was therefore concluded that the process of plasma membrane exfoliation represents a selective rather than a random process. It results in the selective release of certain membrane molecules including certain receptors, tumour antigens (Schirrmacher & Barz, 1986) and degradative enzymes (Kramer *et al.*, 1985). An increased release of IL-2 growth factor receptor by metastatic tumour cells could have a selective growth advantage for the tumour cells because these receptors might absorb the growth factor IL-2 which is required for growth and expansion of host T-lymphocytes with anti-tumour activity. Since the tumour cells themselves are independent in their growth from external IL-2, as shown previously (Diamantstein *et al.*, 1985) this secretion of IL-2 absorbing molecules from the cell surface might interfere with the host response against the tumour and could thus have a selective advantage for the growing tumour cells. The suppression of T-cell responses through competition for T-cell growth factor (IL-2) has been described before (Günther *et al.*, 1982). A selective suppression of cell-mediated autoimmunity has been observed following treatment with anti-IL-2R monoclonal antibodies (Diamantstein & Osawa, 1986).

Further experiments are necessary to elucidate *in vitro* the release of IL-2R by Eb, ESb and ESb-MP cells. It will be of interest to determine whether the released receptor material is membrane bound or whether it is derived perhaps by direct secretion of a variant of the IL-2R which does not bear the transmembrane hydrophobic region that is produced by an independent mRNA lacking this exon of the IL-2 receptor.

The most common human tumour expressing IL-2 receptor is adult T-cell leukaemia (ATL) (Yodoi & Uchiyama, 1986; Waldmann *et al.*, 1984; Yodoi *et al.*, 1983). It will be interesting to study cell-free IL-2R levels in the serum of ATL patients to see whether this might be a useful additional diagnostic and prognostic marker in this disease.

This work was supported in part by the Deutsche Forschungsgemeinschaft (grant Di-152-83).

### References

ALTEVOGT, P., KURNICK, J.T., KIMURA, A.K., BOSSLET, K. & SCHIRRMACHER, V. (1982). Different expression of Lyt differentiation antigens and cell surface glycoproteins by a murine T-lymphoma line and its high metastatic variant. *Eur. J. Immunol.*, **12**, 300.

BARZ, D., GOPPELT, M., SZAMEL, M., SCHIRRMACHER, V. & RESCH, K. (1985). Characterization of cellular and extracellular plasma membrane vesicles from a non-metastasizing lymphoma (Eb) and its metastasizing variant (ESb). I. Biochemical characterization. *Biochim. Biophys. Acta*, **814**, 77.

DIAMANTSTEIN, T., OSAWA, H., GRAF, L. & SCHIRRMACHER, V. (1985). Studies on interleukin 2 receptor expression and IL-2 production by murine T-cell lymphomas. *Br. J. Cancer*, **51**, 23.

DIAMANTSTEIN, T. & OSAWA, H. (1986). The interleukin-2 receptor, its physiology and a new approach to a selective immunosuppressive therapy by anti-interleukin-2 receptor monoclonal antibodies. *Immunol. Rev.*, **92**, 5.

FOGEL, M., ALTEVOGT, P. & SCHIRRMACHER, V. (1983). Metastatic potential severely altered by changes in tumor cell adhesiveness and cell surface sialylation. *J. Exp. Med.*, **157**, 371.

ĞUNTHER, I., HAAS, W. & VON BOEHMER, H. (1982). Suppression of T-cells responses through competition for T-cell growth factor (interleukin 2). *Eur. J. Immunol.*, **12**, 247.

KRAMER, M.D., ROBINSON, P., VLODAVSKY, I., BARZ, D., FRIBERGER, P., FUKS, Z. & SCHIRRMACHER, V. (1985). Characterization of an extracellular matrix-degrading protease derived from a highly metastatic tumour cell line. *Eur. J. Cancer Clin. Oncol.*, **21**, 307.

LARIZZA, L., SCHIRRMACHER, V., GRAF, L., PFLÜGER, E., PERES-MARTINEZ, N. & STÖHR, M. (1984). Suggestive evidence that the highly metastatic variant ESb of the T-cell lymphoma Eb is derived from a spontaneous fusion with a host macrophage. *Int. J. Cancer*, **34**, 699.

MALEK, T.R., ROBB, R.J. & SHEVACH, E.M. (1983). Identification and initial characterization of a rat monoclonal antibody reactive with the murine interleukin 2 receptor-ligand complex. *Proc. Natl Acad. Sci. USA*, **80**, 5694.

MALEK, T., ASHWELL, J.D., GERMAIN, R.N., SHEVACH, E.M. & MILLER, J. (1986). The murine interleukin-2 receptor: Biochemical structure and regulation of expression. *Immunol. Rev.*, **92**, 81.

MURAYAMA, K., LEVERY, S., SCHIRRMACHER, V. & HAKOMORI, S. (1986). Qualitative differences in position of sialylation and surface expression of glycolipids between murine lymphomas with low metastatic (Eb) and high metastatic (ESb) potentials and isolation of a novel disialoganglioside ($GD_{1x}$) from Eb cells. *Cancer Res.*, **46**, 1395.

OSAWA, H. & DIAMANTSTEIN, T. (1984*a*). A rat monoclonal antibody that binds specifically to mouse T-lymphoblasts and inhibits IL-2 receptor functions: A putative anti-IL-2 receptor antibody. *J. Immunol.*, **132**, 2442.

OSAWA, H. & DIAMANTSTEIN, T. (1984*b*). Partial characterization of the putative rat interleukin-2 receptor. *Eur. J. Immunol.*, **14**, 364.

OSAWA, H., JOSIMOVIC-ALASEVIC, O. & DIAMANTSTEIN, T. (1986*a*). Interleukin-2 receptors are released by cells *in vitro* and *in vivo*. I. Detection of soluble IL-2 receptors in cellculture supernatants and in serum of mice by an immunoradiometric assay. *Eur. J. Immunol.*, **16**, 457.

OSAWA, H., JOSIMOVIC-ALASEVIC, O. & DIAMANTSTEIN, T. (1986*b*). Enzyme-linked immunosorbent assay of mouse interleukin-2 receptors. *J. Immunol. Methods*, **92**, 109.

ROBB, R.J., MUNCK, A. & SMITH, K.A. (1981). T-cell growth factor receptors. Quantitation, specificity, and biological relevance. *J. Exp. Med.*, **154**, 1455.

RUBIN, L.A. *et al.* (1985). *Hybridoma*, **4**, 91.

SCHIRRMACHER, V. & JACOBS, W. (1979). Tumor metastases and cell-mediated immunity in a model system in DBA/2 mice. VIII. Expression and shedding of Fc receptors on metastatic tumor cell variants. *J. Supramol. Struct.*, **11**, 105.

SCHIRRMACHER, V., SHANTZ, G., CLAUER, K., KOMITOWSKI, D., ZIMMERMAN, H.-P. & LOHMANN-MATTHES, M.L. (1979*a*). Tumor metastases and cell-mediated immunity in a model system in DBA/2 mice. I. Tumor invasiveness *in vitro* and metastases formation *in vivo*. *Int. J. Cancer*, **23**, 233.

SCHIRRMACHER, V., BOSSLET, K., SHANTZ, G., CLAUER, K. & HÜBSCH, D. (1979*b*). Tumor metastases and cell-mediated immunity in a model system in DBA/2 mice. IV. Antigenic differences between the parental tumor line and its metastasizing variant. *Int. J. Cancer*, **23**, 245.

SCHIRRMACHER, V., FOGEL, M., RUSSMAN, E., BOSSLET, K., ALTEVOGT, P. & BECK, L. (1982). Antigenic variation in cancer metastasis. Immune escape versus immune control. *Cancer Met. Rev.*, **1**, 241.

SCHMIRRMACHER, V. & BARZ, D. (1986). Characterization of cellular and extracellular plasma-membrane vesicles from a low metastasizing lymphoma (Eb) and its high metastatic variant (ESb). II. Inhibitory capacity in cell-cell interaction systems. *Biochim. Biophys. Acta*, **860**, 236.

SCHWARTZ, R., SCHIRRMACHER, V. & MÜHLRADT, P.F. (1984). Glycoconjugates of murine tumor lines with different metastatic capacity. I. Differences in fucose utilization and in glycoprotein patterns. *Int. J. Cancer*, **33**, 503.

SHIMIZU, Ä., KONDO, S., SABE, H., ISHIDA, N. & HONJO, T. (1986). Structure and function of the interleukin-2 receptor: Affinity conversion model. *Immunol. Rev.*, **92**, 103.

UCHIYAMA, T., BRODER, S. & WALDEMANN, T.A. (1981). A monoclonal antibody (anti-Tac) reactive with activated and functionally mature human T-cells. I. Production of anti-Tac monoclonal antibody and distribution of Tac (+) cells. *J. Immunol.*, **126**, 1393.

WALDMANN, T.A., GREENE, W.C., SARIN, P.S., SAXINGER, C., BLAYNEY, D.W. *et al.* (1984). Functional and phenotypic comparison of human T-cell leukemia/lymphoma virus positive adult T-cell leukemia with human T-cell leukemia/lymphoma virus negative Sezary leukemia, and their distribution using anti-Tac. *J. Clin. Invest.*, **73**, 1711.

WALDMANN, T.A. (1986). The structure, function, and expression of interleukin-2 receptors on normal and malignant lymphocytes. *Science*, **232**, 727.

WALLER, C.A., BRAUN, M. & SCHIRRMACHER, V. (1986). Quantitative analysis of cancer invasion *in vitro*: Comparison of two new assays and of tumor sublines with different metastatic capacity. *Clin. Exptl. Metastasis*, **4**, 73.

YODOI, J., UCHIYAMA, T. & MAEDA, M. (1983). T-cell growth factor receptor in adult T-cell leukemia. *Blood*, **62**, 509.

YODOI, J. & UCHIYAMA, T. (1986). IL-2 receptor dysfunction and adult T-cell leukemia. *Immunol. Rev.*, **92**, 135.